# Catalytic Hydrogenation and Heteroatom Removal for the Soluble Organics from Santanghu Bituminous Coal

**DOI:** 10.3390/molecules30040849

**Published:** 2025-02-12

**Authors:** Jia Guo, Guihan Zhao, Akram Naeem, Yaya Ma, Meixia Zhu, Yuan Ren, Wenlong Mo, Xanyong Wei, Xing Fan, Shihao Hao, Ahmad Ali

**Affiliations:** 1 Xinjiang Energy Co., Ltd., Urumqi 830000, China; gj459824057@126.com; 2 State Key Laboratory of Chemistry and Utilization of Carbon Based Energy Resources, Key Laboratory of Coal Clean Conversion & Chemical Engineering Process (Xinjiang Uyghur Autonomous Region), School of Chemical Engineering and Technology, Xinjiang University, Urumqi 830017, Chinafanxing@sdust.edu.cn (X.F.); 3 School of Chemical Engineering, Minhaj University Lahore, Lahore 54000, Pakistan; 4 Hami Quality and Metrology Testing Institute, Hami 839000, China

**Keywords:** Santanghu bituminous coal, soluble organic compounds, catalytic hydrogenation, heteroatom removal, GC-MS

## Abstract

Soluble organics (SBC-L) from Santanghu bituminous coal (SBC) were obtained by extracting the coal with a mixed solvent of CS_2_ and acetone (*v*/*v*′ = 1:1). Catalytic hydrogenation of SBC-L was carried out using isopropanol as the solvent and prepared bimetallic material (Ni-Mo/γ-Al_2_O_3_) as the catalyst, and the hydrogenation product (SBC-L_IP320_) was obtained. Gas chromatography-mass spectrometry (GC-MS) was used to compare the difference in the composition and distribution of SBC-L and SBC-L_IP320_; thus, the effect of the used catalyst on the hydrogenation performance and heteroatom removal of SBC-L can be investigated. Results showed that the organic compounds in SBC-L and SBC-L_IP320_ could be classified into aliphatic hydrocarbons (AHS), arenes, oxygen-containing organic compounds (OCOCs), nitrogen-containing organics (NCOCs), and compounds containing other heteroatoms (OHACOCs). The relative contents of AHS and arenes detected in SBC-L_IP320_ were higher than those of SBC-L, while the contents of OCOCs, NCOCs, and OHACOCs decreased, and no S-containing compounds could be detected in SBC-L_IP320_. It can be concluded that the prepared catalyst presents good de-oxygenation, de-sulfurization, de-nitrogenation, and hydrocracking performance.

## 1. Introduction

Coal hydrogenation liquefaction technology is one of the important technologies to solve China’s future energy issues. This technology can achieve a high conversion rate and oil yield under mild conditions, providing a new way for the efficient graded conversion and utilization of low-rank coal [1,2,3]. The Santanghu coalfield is an important coal production and processing base in the Hami region of Xinjiang, China, and the coal type is mainly long-flame coal (one of the low-rank coals), with the coalification degree slightly higher than that of lignite. How to remove coal-based soluble heteroatom-containing organic compounds, such as various forms of heteroatoms (O, N, and S) in coal-based liquid fuel, has become one of the keys to the efficient utilization of low-rank coal [4,5].

Solvent extraction is an effective method for obtaining soluble organic compounds from coal, which makes it possible to transition low-rank coal from utilization characterized as “high pollution, low efficiency, and low added value” to one that is “clean, efficient, and high in added value” [6,7,8]. Ya-Ya Ma et al. [9] used petroleum ether, methanol, carbon disulfide, and other solvents to perform sequential five-stage extraction for Hefeng (Xinjiang) sub-bituminous coal. Results showed that methanol gave a higher extraction rate, while the isometric carbon disulfide/acetone mixture solvent was more conducive to the dissolution and diffusion of alcohol compounds. Catalytic hydrogenation deoxygenation of isopropanol-soluble organic compounds from Dongming lignite was also investigated by this group. Results showed that the Co-Mo/γ-Al_2_O_3_ catalyst for the conversion of soluble compounds could achieve the effects of hydrogenation, hydrocracking, and heteroatom removal [10].

In the process of coal hydrogenation, the catalyst plays an essential role. On the one hand, catalysts can improve reaction rate and reduce reaction temperature and pressure. On the other hand, the addition of appropriate catalysts can remove some heteroatoms (S and N) to reduce environmental pollution. More importantly, the addition of a catalyst can improve the cracking and conversion of coal to obtain high-added-value liquid fuel oils and chemical products [11,12]. Common coal hydrogenation catalysts include metal catalysts, metal oxide catalysts, metal sulfide catalysts, acidic catalysts, and basic catalysts, etc. [13,14,15,16,17,18]. Efficient and low-cost catalysts are conducive to the removal of heteroatoms in the conversion of low-rank coal. Qi S C et al. [19] used an in situ de-composition method to load carbonyl nickel onto an HZSM-5 carrier to prepare a highly active Ni/HZSM-5 catalyst, which was used for deep hydrogenation and removal of heavy aromatic hydrocarbons (HAs) from coal tar to obtain clean liquid fuel. Kang Y H et al. [20] synthesized a nickel-based Y/ZCM-5 catalyst and applied it to the catalytic hydrogenation of Hecaogou sub-bituminous coal, resulting in clean liquid fuel, primarily composed of alkanes, alkenes, and hydrogenated aromatics. Li W T et al. [21] used prepared Ni/Z5A catalyst for the catalytic hydrogenation of methanol-soluble organic compounds from Xiaolongtan lignite, and it is found that the catalyst played a dual role in breaking >CH-O- and hydrogenating aromatics.

The above results indicate that by obtaining soluble organic compounds through extraction or thermal dissolution, followed by catalytic hydrogenation and heteroatom removal of the soluble organics, it is possible to produce clean liquid fuels, high-density fuels, base oils for high-grade lubricants, as well as high value-added chemicals. However, how to enhance the efficiency of the extraction and catalytic hydrogenation process to obtain clean fuels or value-added chemicals warrants further exploration.

In this work, soluble organic compounds (SBC-L) from Santanghu bituminous coal (SBC) were obtained by extracting the coal with a mixed solvent of CS_2_ and acetone (*v*/*v* = 1:1). Low-boiling-point and low-viscosity isopropanol was selected as the hydrogenation solvent, and gas chromatography-mass spectrometry (GC-MS) was used to compare the difference in the composition and distribution of soluble organic matter (SBC-L) and its hydrogenation products (SBC-LIP_320_). Thus, the influence of prepared Ni-Mo/γ-Al_2_O_3_ bimetallic catalyst on the catalytic hydrogenation of soluble organic matter, especially the removal of heteroatoms, could be explored.

## 2. Materials and Methods

### 2.1. Materials

The coal sample (SBC) was selected from the Santanghu coal mine in Hami, Xinjiang, China. After crushing, it was sifted through a 200-mesh sieve (<75 μm) and dried naturally for 24 h. Proximate and ultimate analyses of SBC are shown in Table 1 [22]. All the solvents are analytical reagents and are distilled in a rotary evaporator before use.

### 2.2. SBC-L from SBC

Under ultrasonic conditions, a mixed solvent of carbon disulfide (CS_2_) and acetone (*v*/*v*′ = 1:1) is used to extract and separate the easily soluble organic components from the coal sample, resulting in the extraction yield of 5.05%.

### 2.3. Preparation of Ni-Mo/γ-Al_2_O_3_ Bimetallic Catalyst

**Figure 1:**
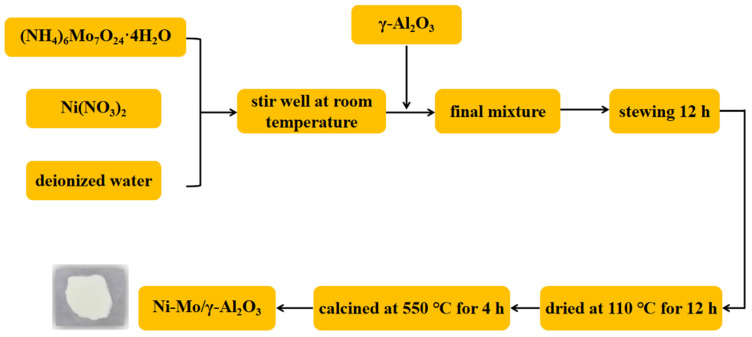
Preparation process of the catalyst.

As shown in Figure 1, a mixed solution was formed by dissolving (NH_4_)_6_Mo_7_O_24_·4H_2_O and Ni(NO_3_)_2_ (Ni:Mo = 1:2.4) in an appropriate amount of deionized water using the impregnation method. γ-Al_2_O_3_ was added with Ni/Mo loading of 10 wt.%, standing for 12 h, dried at 110 °C for 12 h, and calcined in a tube furnace at 550 °C for 4 h to obtain Ni-Mo/γ-Al_2_O_3_ bimetallic catalyst.

### 2.4. Catalytic Hydrogenation

**Figure 2:**
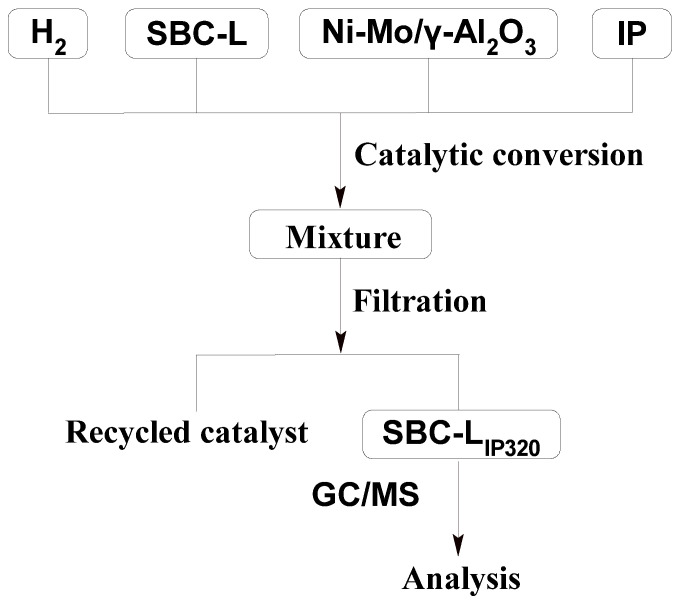
Catalytic hydrogenation process for SBC-L.

As shown in Figure 2, 2 g of dried SBC-L, 0.2 g of Ni-Mo/γ-Al_2_O_3_ catalyst, and 20 mL of isopropanol (IP) were added into the autoclave. After purging the autoclave with H_2_ to displace air three times, the autoclave was heated to 320 °C under an initial H_2_ pressure of 0.4 MPa and maintained for 2 h. Upon completion of the hydrogenation process, the autoclave was cooled to room temperature, and the mixture was filtered to separate into filtrate and residue. The residue was washed repeatedly with IP until the filtrate became colorless. The used solvent was recycled by rotary evaporation, and the hydrogenation products would also be obtained, which is labeled as SBC-L_IP320_.

### 2.5. Analytical Methods

The products SBC-L and SBC-L_IP320_ were analyzed using Agilent 7890A/5795C GC/MS. The instrument was equipped with a DB-17MS (30.0 m × 250 μm, 0.25 μm) chromatographic column, and high-purity helium gas (1 mL/min) was used as the carrier gas. The inlet initial temperature was set as 60 °C for 3 min, followed by a ramp rate of 10 °C/min up to 300 °C for 10 min. The split ratio was 20:1, and the mass scan range was set from 30 to 500 amu.

## 3. Results and Discussion

### 3.1. Composition Characteristics of SBC-L

**Figure 3:**
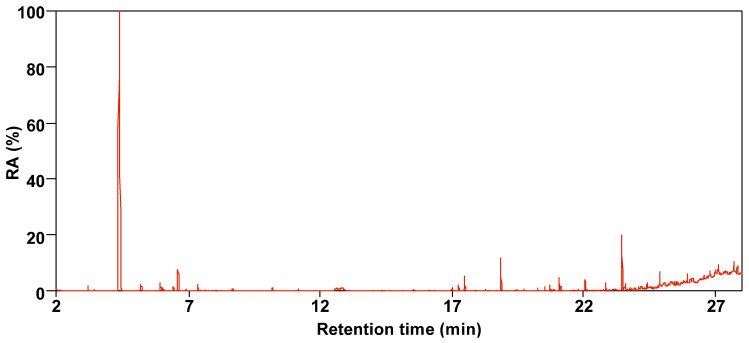
Total ion chromatogram of SBC-L.

**Figure 4:**
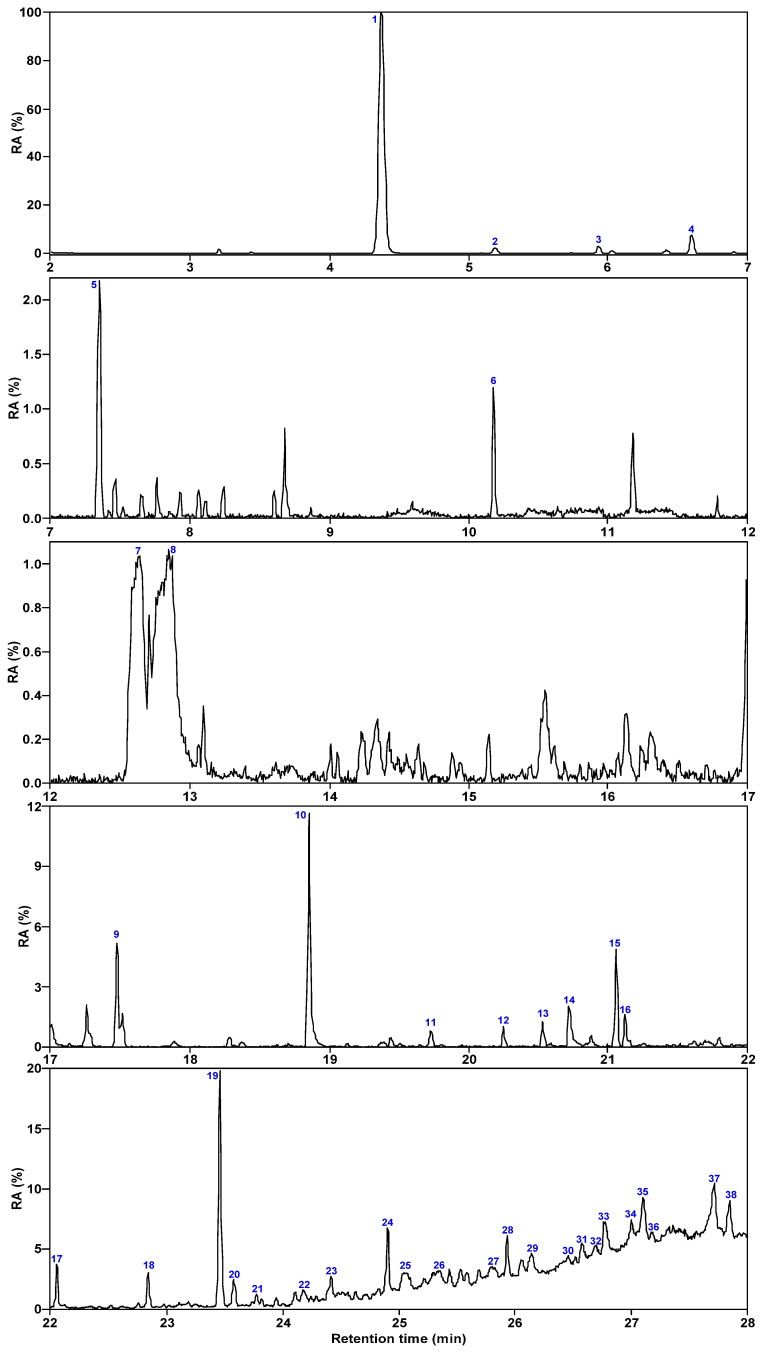
Relative content of each component in SBC-L.

Figure 3 and Figure 4 show the total ion chromatogram of SBC-L, with a retention time of 2–28 min selected. The number of compounds detected in SBC-L by GC/MS is 38, which can be divided into 5 categories: alkanes, aromatics, oxygen-containing organic compounds (OCOCs), nitrogen-containing compounds (NCOCs), and other heteroatomic-containing organics (OHACOCs).

Table 2 lists all the organic compounds detected in SBC-L along with their relative contents. SBC-L mainly contains 2 types of alkanes, 13 types of aromatic hydrocarbons, 13 types of oxygen-containing organic compounds, 8 types of nitrogen-containing compounds, and 2 types of organics with other heteroatoms. Among them, aromatic hydrocarbons are primarily dominated by polycyclic aromatics (relative content of 9.48%), followed by monocyclic ones (6.16%). The relative content of NCOCs (15.07%) is high, and nitrogen in these compounds is present in the form of C-N bonds. And OHACOCs (3.61%) are low in content with sulfur-containing compounds dominating.

**Figure 5:**
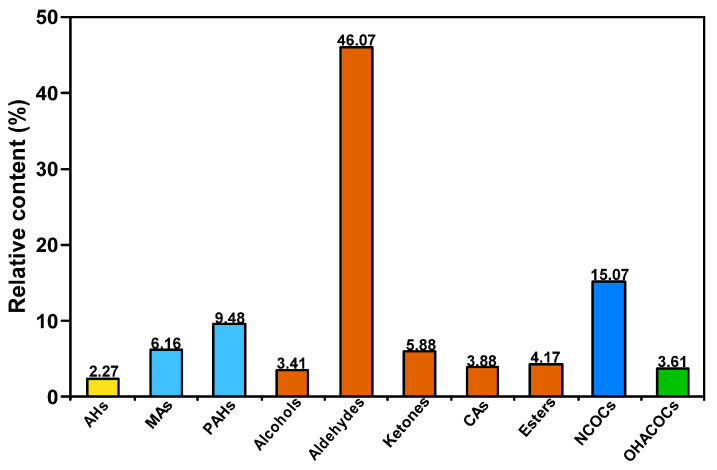
Distribution of group composition in SBC-L.

Figure 5 presents the distribution of the group composition of SBC-L. As can be seen from the figure, SBC-L is predominantly composed of oxygen-containing compounds with a relative content of 63.41%, followed by aromatic hydrocarbons (15.64%). Among them, the oxygen-containing organics mainly include alcohols, aldehydes, ketones, esters, and carboxylic acids, with aldehydes being the dominant group (46.07%).

### 3.2. Composition Characteristics of SBC-L_IP320_

**Figure 6:**
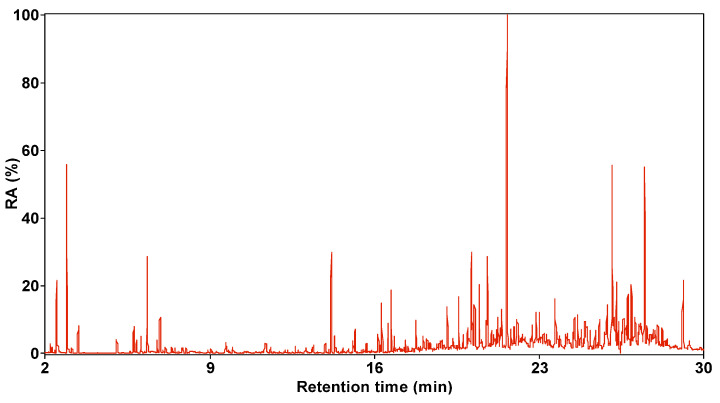
Total ion chromatogram of SBC-L_IP320_.

**Figure 7:**
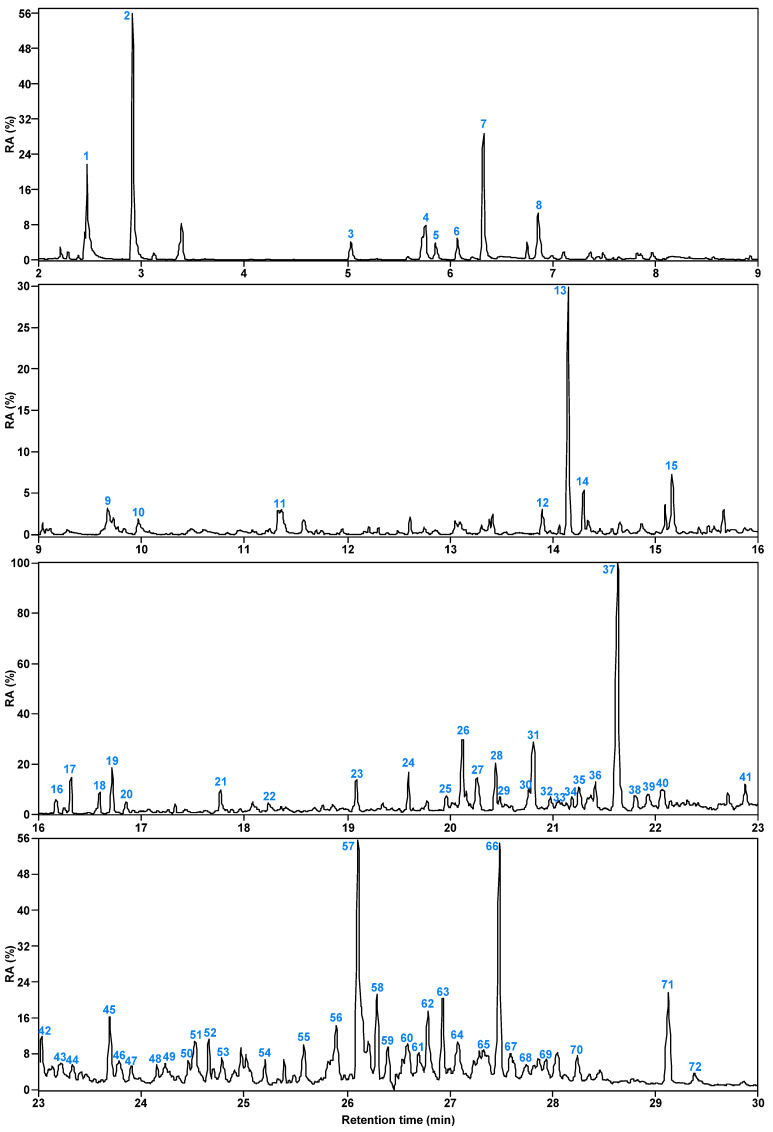
Relative content of each compound in SBC-L_IP320_.

Figure 6 and Figure 7 present the total ion flow chromatograms of SBC-L_IP320_ with a retention time of 2–30 min. Through GC/MS analysis, 72 organic compounds were identified in SBC-L_IP320_, including alkanes, aromatic hydrocarbons, OCOCs, and NCOCs.

Table 3 lists the organic compounds detected in SBC-L_IP320_ along with their relative contents. According to the table, SBC-L_IP320_ contains 11 alkanes, 26 aromatic hydrocarbons, 29 OCOCs, and 6 NCOCs. Among them, hydrocarbons are primarily alkenes with a relative content of 14.91%, followed by alkanes (4.14%); aromatic hydrocarbons are mainly polycyclic aromatics (25.44%), followed by monocyclic ones (3.83%).

**Figure 8:**
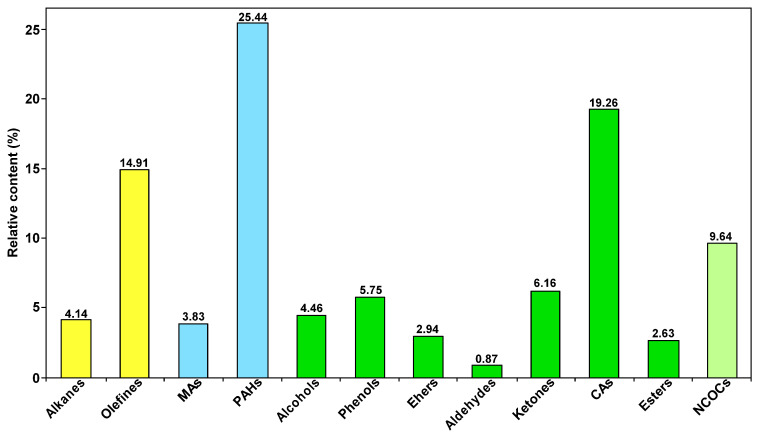
Distribution of group composition in SBC-L_IP320_.

Figure 8 provides the distribution of the group composition of SBC-L_IP320_. As can be seen from the figure, OCOCs are predominant in SBC-L_IP320_ (relative content of 42.07%), followed by aromatic hydrocarbons (29.27%). The OCOCs group includes alcohols, phenols, ethers, aldehydes, ketones, esters, and carboxylic acids, with carboxylic acids (19.26%) being the higher content group.

**Figure 9:**
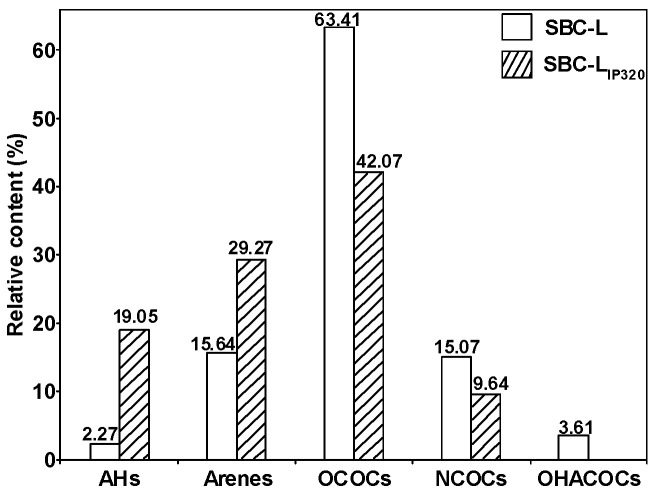
Distribution of group compositions for SBC-L and SBC-L_IP320_.

Figure 9 presents the distributions of the group composition of SBC-L and its catalytically hydrogenated products, SBC-L_IP320_. As shown in the figure, compared to SBC-L, the contents of alkanes and aromatic hydrocarbons detected in SBC-L_IP320_ increased, while the contents of OCOCs, NCOCs, and OHACOCs decreased, indicating that the catalyst of Ni-Mo/γ-Al_2_O_3_ might improve side-chain cracking and ring-opening reactions, increasing the contents of alkanes and arenes. The content of OCOCs shows the most significant change, indicating that Ni-Mo/γ-Al_2_O_3_ could effectively break C-O and/or C=O bonds. Thus, catalytic hydrogenolysis and hydrogenative deoxygenation and denitrogenation can simultaneously convert the oxygen- and nitrogen-containing organic compounds (in SBC-L) into other products (alkanes and arenes) and effectively remove the heteroatoms.

**Figure 10:**
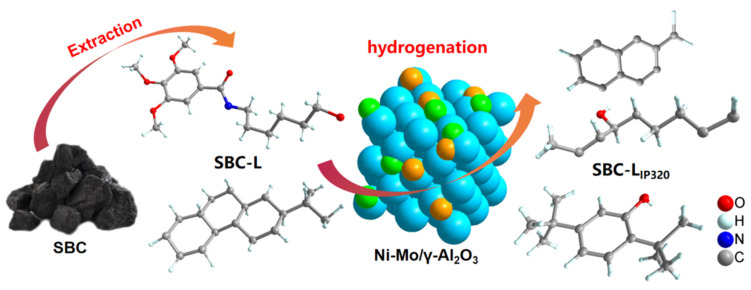
Schematic diagram of the catalytic hydrogenation process by Ni-Mo/γ-Al_2_O_3_.

The scheme of the catalytic transformation process for SBC-L is shown in Figure 10. For compounds containing one or more oxygen atoms, the cleavage of carbon–oxygen and carbon–carbon bonds can induce the formation of new compounds, which may be one of the reasons for the increase in the content of aromatic hydrocarbons. At the same time, the low initial hydrogen pressure (0.4 MPa) might be beneficial for the breakage of bridge bonds between aromatic rings, contributing to the increase in arenes.

**Figure 11:**
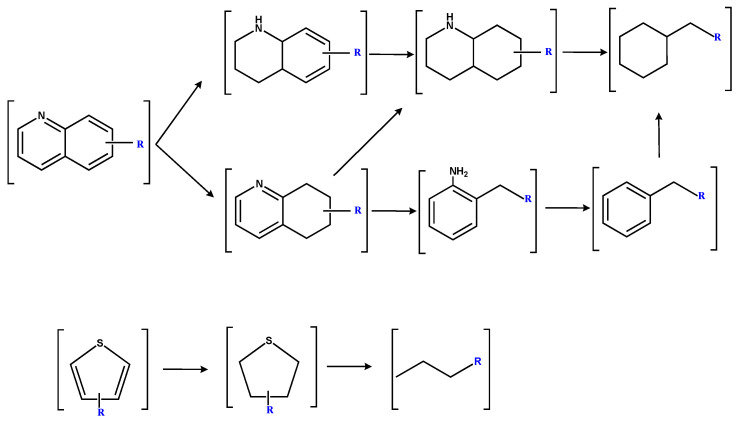
Possible hydrogenation pathways for quinoline and thiophene groups in SBC-L [22,23,24].

Figure 11 illustrates the potential catalytic hydrogenation pathways for quinoline and thiophene groups in SBC-L. The quinoline group primarily undergoes transformation through two possible pathways [23]. One pathway involves the initial hydrogenation of the nitrogen ring in quinoline, followed by an aromatic ring, and followed by ring-opening and removal of the nitrogen atom, resulting in the formation of alkanes. Other pathways involve firstly the hydrogenation of the aromatic ring, followed by its opening, after which the nitrogen ring is hydrogenated, and the nitrogen-containing bond, C-N, is broken to produce aromatic hydrocarbons. Further hydrogenation in this process could lead to the formation of alkanes. The catalytic hydrogenation process of the thiophene group might probably begin with a hydrogenation process [24,25], followed by the cleavage of the C-S bond, leading to the formation of alkanes and H_2_S resulting in the increase in alkanes in SBC-L_IP320_.

## 4. Conclusions

In this paper, soluble compounds (SBC-L) from Santanghu bituminous coal (SBC) were extracted with a mixed solvent, and its catalytic hydrogenation products (SBC-L_IP320_) were obtained by the Ni-Mo/γ-Al_2_O_3_ catalyst. Both SBC-L and SBC-L_IP320_ are primarily composed of alkanes, aromatic hydrocarbons, oxygen-containing compounds (OCOCs), nitrogen-containing organics (NCOCs), and other heteroatom-containing compounds (OHACOCs). Compared to SBC-L, the content of alkanes and aromatic hydrocarbons detected in SBC-L_IP320_ increased, which may be derived from the ability of the Ni-Mo/γ-Al_2_O_3_ catalyst to hydrogenate the bridge bonds between aromatic rings, leading to the formation of alkanes and arenes. Meanwhile, the relative content of OCOCs, NCOCs, and OHACOCs decreased obviously, and no sulfur-containing compounds were detected, suggesting that the Ni-Mo/γ-Al_2_O_3_ catalyst can effectively remove oxygen, sulfur, and nitrogen atoms, presenting a good impurity removal effect. The obtained SBC-L_IP320_ has the potential to be used in the production of clean liquid fuels, high-grade lubricant base oils, and high-value chemicals. However, further research is needed to explore more efficient methods for producing high-purity and high-value chemicals.

## Figures and Tables

**Table 1:** Proximate and Ultimate analyses (wt.%) of SBC.

Sample	Proximate Analysis				Ultimate Analysis					H/C
	M_ad_	A_ad_	VM_ad_	FC_ad_	C_daf_	H_daf_	O_daf_ *	N_daf_	S_t,d_	
SBC	9.48	5.81	26.59	58.12	69.10	3.39	>25.88	0.91	0.72	0.59

ad: air dry base; *: by difference subtraction calculation; daf: dry ash free base.

**Table 2:** Organic compounds detected in SBC-L by GC/MS.

Species	Peak	Compound	CAS	RC(%)
AHS	7	Cyclobutene, bis(1-methylethylidene)-	003642-14-6	1.04
	9	2-Phenyl-1,3-cyclohexadiene	015619-34-8	1.23
Monocyclic aromatics (MAs)	2	Benzene, 1-ethyl-3-methyl-	000620-14-4	0.95
	3	Benzene, 1,2,4-trimethyl-	000095-63-6	0.95
	4	Benzene, 1,2,3-trimethyl-	000526-73-8	2.46
	5	Mesitylene	000108-67-8	0.66
	15	Benzene, 1,2,3,4-tetramethyl-4-(1-methylethenyl)-	061142-76-5	1.14
Polycyclic aromatic hydrocarbons (PAHs)	11	Anthracene	000120-12-7	0.19
	16	Pyridine, 3-(2,4,6-trimethylphenyl)-	075601-34-2	0.38
	18	Fluoranthene	000206-44-0	0.85
	19	Retene	000483-65-8	5.31
	20	Pyrene	000129-00-0	0.76
	21	11H-Benzo[a]fluorene	000238-84-6	0.38
	22	Pyrene, 1-methyl-	002381-21-7	0.57
	31	Chrysene	000218-01-9	1.04
Alcohols	23	1-Pyrenemethanol	024463-15-8	0.76
	28	Hexadeca-2,6,10,14-tetraen-1-ol, 3,7,11,16-tetramethyl-	007614-21-3	1.23
	36	2-[2-Quinolyl] methylene quinuclidin-3-ol	1000256-36-9	1.42
Aldehydes	1	2-Pentanone, 4-hydroxy-4-methyl-	000123-42-2	46.07
Ketones	17	Acetophenone, 4,4′-methylene	000790-82-9	0.85
	25	6-Tridecanone	022026-12-6	1.52
	35	1,2-Dihydropyrido(3,2,1-kl)phenothiazin-3-one	069513-42-4	3.51
Carboxylic acids (CAs)	10	n-Decanoic acid	000334-48-5	2.94
	13	Benzenebutanoicacid, 2-carboxy-.gamma.-oxo-	027415-09-4	0.28
	14	Tetradecanoic acid	000544-63-8	0.66
Esters	24	Dicyclohexyl phthalate	000084-61-7	1.80
	29	Isonipecotic acid, N-isobutoxycarbonyl-, heptyl ester	1000322-06-6	0.95
NCOCs	6	3-Acetyl-2-oxo-1,3-oxazolidine	001432-43-5	0.28
	12	Benzoic acid, 2-phenylhydrazide	000532-96-7	0.19
	26	[1,1′-Biphenyl]-4-carbonitrile, 4′-propoxy-	052709-86-1	0.95
	27	Mirtazapine	085650-52-8	1.04
	30	Benzamide, 2,3-dinitro-	019613-80-0	1.14
	33	9-Octadecenamide, (Z)-	000301-02-0	2.94
	34	2H-1,4-Benzodiazepin-2-one, 7-amino-1,3,4,5-tetrahydro-5-phenyl-	1000362-81-4	3.03
	37	Benzamide, N-cyclododecyl-3,4,5-trimethoxy-	1000311-05-7	5.50
OHACOCs	8	1,3-Dithiolane, 2,2-dimethyl-	006008-78-2	1.52
	38	2-[2-Thienyl]-4-acetyl quinoline	027302-83-6	2.09

**Table 3:** Organic compounds detected in SBC-L_IP320_ by GC/MS.

Species	Peak	Compound	CAS	RC (%)
Alkanes	17	Pentadecane, 2,6,10,14-tetramethyl-	001921-70-6	0.93
	19	Cyclopentane, ethyl-	001640-89-7	1.22
	34	Pentadecane	000629-62-9	0.52
	50	Heptadecane	000629-78-7	0.64
	54	Hexadecane	000544-76-3	0.83
Olefins	5	Benzene, (1-methylethyl)-	000098-82-8	0.29
	18	4-Nonene	002198-23-4	0.66
	32	Benzo[b]naphtho[2,3-d]furan	000243-42-5	0.87
	37	Retene	000483-65-8	9.59
	41	6,6-Diphenylfulvene	002175-90-8	1.72
	51	9-Tricosene, (Z)-	027519-02-4	1.78
Monocyclic aromatics (MAs)	3	Benzene, 1-ethyl-3-methyl-	000620-14-4	0.31
	6	Benzene, 1-ethyl-4-methyl-	000622-96-8	0.35
	7	Benzene, 1,2,3-trimethyl-	000526-73-8	1.97
	8	Benzene, 1,2,4-trimethyl-	000095-63-6	0.89
	14	1H-Indene, 2,3-dihydro-1,1,2,3,3-pentamethyl-	001203-17-4	0.31
Polycyclic aromatic hydrocarbons (PAHs)	9	Naphthalene	000091-20-3	0.39
	15	Naphthalene, 2,3,6-trimethyl-	000829-26-5	0.58
	16	Naphthalene, 1,6-dimethyl-4-(1-methylethyl)-	000483-78-3	0.42
	20	1,4,5,8-Tetramethylnaphthalene	002717-39-7	0.39
	26	7-Isopropyl-1,1,4a-trimethyl-1,2,3,4,4a,9,10,10a-octahydrophenanthrene	1000210-28-9	2.24
	27	Pyrene	000129-00-0	1.64
	28	7-Butyl-1-hexylnaphthalene	055000-55-0	1.43
	30	Fluoranthene	000206-44-0	0.98
	38	Pyrene, 1-methyl-	002381-21-7	0.93
	39	1,2,3,4-Tetrahydrobenz[a]anthracene	004483-98-1	1.35
	40	1-benzyl-3-methylnaphthalene	1000379-93-5	1.47
	42	8-Isopropyl-1,3-dimethylphenanthrene	135886-06-5	1.16
	43	Benzo[ghi]fluoranthene	000203-12-3	1.22
	45	Benz[a]anthracene	000056-55-3	1.60
	46	Triphenylene	000217-59-4	1.06
	49	Benz[a]anthracene, 5-methyl-	002319-96-2	1.27
	61	Benzo[a]pyrene	000050-32-8	1.33
	62	1,1′:3′,1″:3″,1‴:3‴,1⁗-Quinquephenyl	016716-13-5	2.18
	65	13H-Dibenzo[a,h]fluorene	000239-85-0	1.76
	70	1,1′:2′,″-Terphenyl,3′,4′-dimethyl-5′,6′-diphenyl-	020143-37-7	1.06
	72	Benzo[ghi]perylene	000191-24-2	0.98
Alcohols	25	4,4,5,8-Tetramethylchroman-2-ol	082391-05-7	0.62
	36	Isopropyl stearate	000112-10-7	1.06
	64	2R-2,8-dimethyl-2-(4,8,12-trimethyltridecyl)-3,4-dihydro-2H-1-benzopyran-6-ol	000119-13-1	1.76
	68	.beta.-Tocopherol	000148-03-8	1.02
Phenols	11	Phenol, m-tert-butyl-	000585-34-2	0.52
	13	Phenol, 2,5-bis(1,1-dimethylethyl)-	005875-45-6	1.83
	69	Cannabidiol	013956-29-1	0.95
	71	Phenol,4,4′-methylenebis[2,6-bis(1,1-dimethylethyl)-	000118-82-1	2.45
Ethers	44	1,1′-Biphenyl, 2-phenoxy-	006738-04-1	0.68
	47	Benzo[2,1-b:3,4-b′]bisbenzofuran	000222-23-1	0.79
	60	Dinaphtho[2,1-b:1′,2′-d]furan	000194-63-8	1.47
Aldehydes	33	9-Ethyl-10-methylanthracene	019713-49-6	0.87
Ketones	2	3-Penten-2-one, 4-methyl-	000141-79-7	3.61
	4	Acetophenone	000098-86-2	0.85
	12	Ethanone,1-[4-(1-methyl-2-propenyl)phenyl]-	109586-49-4	0.21
	67	4,6-Dimethyl-4′-hydroxy-2-benzylidene-coumaran-3-one	002567-73-9	1.49
Carboxylic acids (CAs)	22	4′-Ethyl-4-biphenylcarboxylic acid	005731-13-5	0.56
	24	n-Hexadecanoic acid	000057-10-3	1.08
	35	6-Octadecenoic acid, (Z)-	000593-39-5	1.14
	52	Docosanoic acid	000112-85-6	0.79
	57	Tetracosanoic acid	000557-59-5	5.79
	58	Phthalic acid, 3,4-dimethylphenyl 3,5-dimethylphenyl ester	1000315-70-6	1.87
	59	Phthalic acid, di(2,3-dimethylphenyl) ester	1000357-09-2	1.10
	63	Phthalic acid, 3,4-dimethylphenyl 3,5-dimethylphenyl ester	1000315-70-6	1.85
	66	Hexacosanoic acid	000506-46-7	5.08
Esters	10	Benzoic acid, 1-methylethyl ester	000939-48-0	0.23
	23	Phthalic acid, butyl isohexyl ester	1000309-03-6	1.24
	29	Benzoic acid, 6-formyl-2,3-dimethoxy-, ethyl ester	1000196-80-7	0.54
	48	Phthalic acid, monocyclohexyl ester	007517-36-4	0.62
NCOCS	1	Benzonitrile, 4-hydroxy-	000767-00-0	1.99
	21	4-Methyl-3,6-decandione	1000374-13-2	0.95
	31	10H-Benzo[b][1,8]naphthyridin-5-one, 7-ethyl-2,4-dimethyl-	343780-17-6	2.39
	53	2,2′-Biquinoline	000119-91-5	0.97
	55	Benzo[f]quinoline, 5,6-dihydro-3-phenyl-	022877-22-1	1.31
	56	13-Docosenamide, (Z)-	000112-84-5	2.03

## Data Availability

The original contributions presented in this study are included in the article. Further inquiries can be directed to the corresponding author(s).

## Nomenclature

RC	Relative content
CHC	Catalytic hydrogenation
AHs	Aliphatic hydrocarbon
MAs	Monocyclic aromatics
PAHs	Polycyclic aromatic hydrocarbon
CAs	Carboxylic acids
OCOCs	Oxygenated compounds
NCOCs	Nitrogen-containing organic compounds
OHACOCs	Compounds containing other heteroatoms
